# Robot-assisted approach versus open surgery and conventional laparoscopy for radical prostatectomy for prostate cancer: a micro-costing study

**DOI:** 10.1186/s13561-025-00652-5

**Published:** 2025-07-05

**Authors:** Sophie Bouvet, Sihame Chkair, Jean Pierre Daurès, Sarah Kabani, OptiPRobot Study Group, Anne Damman, Gregoire Poinas, Sandy Lacombe, Marie Paule Bourbon, Renaud Lardon, Anne Claire Masson, Sylvie Tilhet Chassaigne, Jean Baptiste Roche, Laurence Ponroy, Xavier Cathelineau, Thomas Pignier, Thierry Chevallier, Eric Lechevallier, Stéphane Droupy

**Affiliations:** 1 Department of Biostatistics, Clinical Epidemiology, Public Health and Innovation in Methodology (BESPIM), CHU Nîmes, Univ Montpellier, 30029 Nîmes, France; 2 Department of Urology, Andrology and Sexology, CHU Nîmes, Univ Montpellier, Nîmes, France; 3 UMR 1302, Institute Desbrest of Epidemiology and Public Health, INSERM, Univ Montpellier, Montpellier, France; 4 Languedoc Mutual Group – Hospital and Care Division, Montpellier, France; 5 Department of Urology and Renal Transplantation, Assistance Publique-Hôpitaux de Marseille, Aix-Marseille Université, Marseille, France

**Keywords:** Micro-costing study, Open surgery, Private hospital, Public hospital, Radical prostatectomy, Robot assisted surgery, Cost analysis

## Abstract

**Background:**

The economic impact of RARP versus laparoscopic (LRP) or open surgical radical prostatectomy (OSRP) is unclear. The objective is to estimate and compare the total cost of radical prostatectomy with and without robot assistance from the French establishment perspective. This estimate can assess the cost benefit of robotic-assisted radical prostatectomy (RARP) and determine who should pay.

**Methods:**

A micro-costing bottom-up time-and-motion approach was used based on 2018 prices (€). In public hospitals, observed data for OSRP and RARP was used; in private hospitals, expert opinions were sought from clinicians for RARP and LRP. Average costs, costs per minute of surgery and costs per expenditure were compared between techniques. A sensitivity analysis accounted for variability in cost of personnel and amortized cost of Da Vinci robot.

**Results:**

The average estimated cost of surgery was 4683.35€ [95% CI=2900; 6467.2] more for RARP versus LRP in private clinics, and 3744€ [95% CI=3525; 3963] for RARP versus OSRP in public hospital. Recovery costs were equivalent between techniques (112.9€ for RARP and LRP in private and 46.1€ [95% CI=31.8; 60.4] for OSRP and 47.8€ [95% CI=39.1; 56.5] for RARP in public hospital). The sensitivity analysis confirmed the extra cost for RARP versus LRP or OSRP.

**Conclusions:**

Depending on the surgery compared (OSRP or LRP), institute type (public or private) and data source (observed or expert opinion), the extra cost of the robot varied from 3744€ to 4683.35€. The amortized cost of the robot and its specific materials were the main elements of the difference.

**Trial registration:**

This comparative, multi-centre economic study combines one secondary objective from the RoboProstate study (NCT01577836) and part 1 of the OptiPRobot study (IRB #19.07.03).

**Supplementary Information:**

The online version contains supplementary material available at 10.1186/s13561-025-00652-5.

## Background

Around 20,0000 radical prostatectomies (RP) are performed annually in France (PMSI databases), with open surgical radical prostatectomy (OSRP) being replaced by laparoscopic radical prostatectomy (LRP). The overall and progression-free survival of robotic-assisted radical prostatectomy (RARP) versus OSRP and conventional LRP [1, 2] is ill defined and the superiority or non-inferiority of RARP over existing techniques is not fully established [2, 3].

There are more than 6700 Da Vinci robots (Intuitive Surgical, Sunnyvale, CA, USA) worldwide [4] and their use is growing in many surgical indications. However, the robot costs 1.5-2.5 million euros depending on the model and the conditions negotiated with the supplier, plus 10% for annual maintenance and 1300€−2500€ for instruments attached to the robot, which have a 10-intervention lifespan [5]. In France, even RP generates the same French Diagnosis Related Group (DRG) to the care provider. This fee may differ according to the severity but not according to surgical technique and the additional cost of RARP is usually absorbed by the hospital. However, in private clinics, it may sometimes be transferred to patients, which makes the distinction between public and private institutions relevant when considering the financial impact on patients.

Although some studies suggest that robot-assisted radical prostatectomy may offer improved functional outcomes—such as better preservation of continence and sexual potency—compared to conventional techniques, these results remain heterogeneous and inconclusive. In its 2016 report, the French National Authority for Health (HAS) conducted a critical review of the available evidence and concluded that there is insufficient high-quality data to establish the superiority or non-inferiority of robotic-assisted surgery, particularly in terms of oncological and functional outcomes. In this context, economic evaluation becomes essential to support decision-making regarding the adoption and diffusion of this costly technology.

Thus, this study aimed to estimate and compare the cost of RP according to technique to deduce the additional cost with the Da Vinci robot. This will generate a reliable estimate of the cost in the operating and recovery room for comparative studies. Many studies use gross costing data from healthcare institutions or DRGs [6–14], but this cannot estimate the real difference in cost [15]. A few micro-costing studies [16–20] have estimated the real cost of the procedure. Micro-costing is unsuitable for large series of patients and centres, limiting representativeness [21]. However, as RP is standardised across techniques, procedure cost can be estimated with relatively few observations. Moreover, it is important to identify the levers of cost differences between techniques to: (i) target the data to collect (ii) optimise robot deployment considering the costs to be reduced.

## Methods

### Study design

This comparative, multi-centre economic study combines one secondary objective from the RoboProstate (NCT01577836) prospective and observational study and part 1 of the OptiPRobot study (IRB #19.07.03).

This objective focuses exclusively on the cost of the surgical procedure itself, providing a detailed estimate of the additional cost incurred by healthcare institutions for robot-assisted surgery. Other relevant cost components—such as hospital stay and follow-up care—are reimbursed separately by the national health insurance and thus fall outside the scope of this analysis. These aspects, along with the long-term cost implications, are being addressed in a complementary study currently underway. The RoboProstate study was approved by the local ethics committee (#2011.12.09 sept) and all patients provided signed informed consent. Data from the OptiPRobot study were treated according with CNIL (Commission Nationale de l'Informatique et des Libertés; MR004) and the General Data Protection Regulation (GDPR). The RoboProstate study compared RARP versus OSRP in public hospitals, whereas OptiPRobot compared RARP versus LRP in private hospitals.

**Fig. 1:**
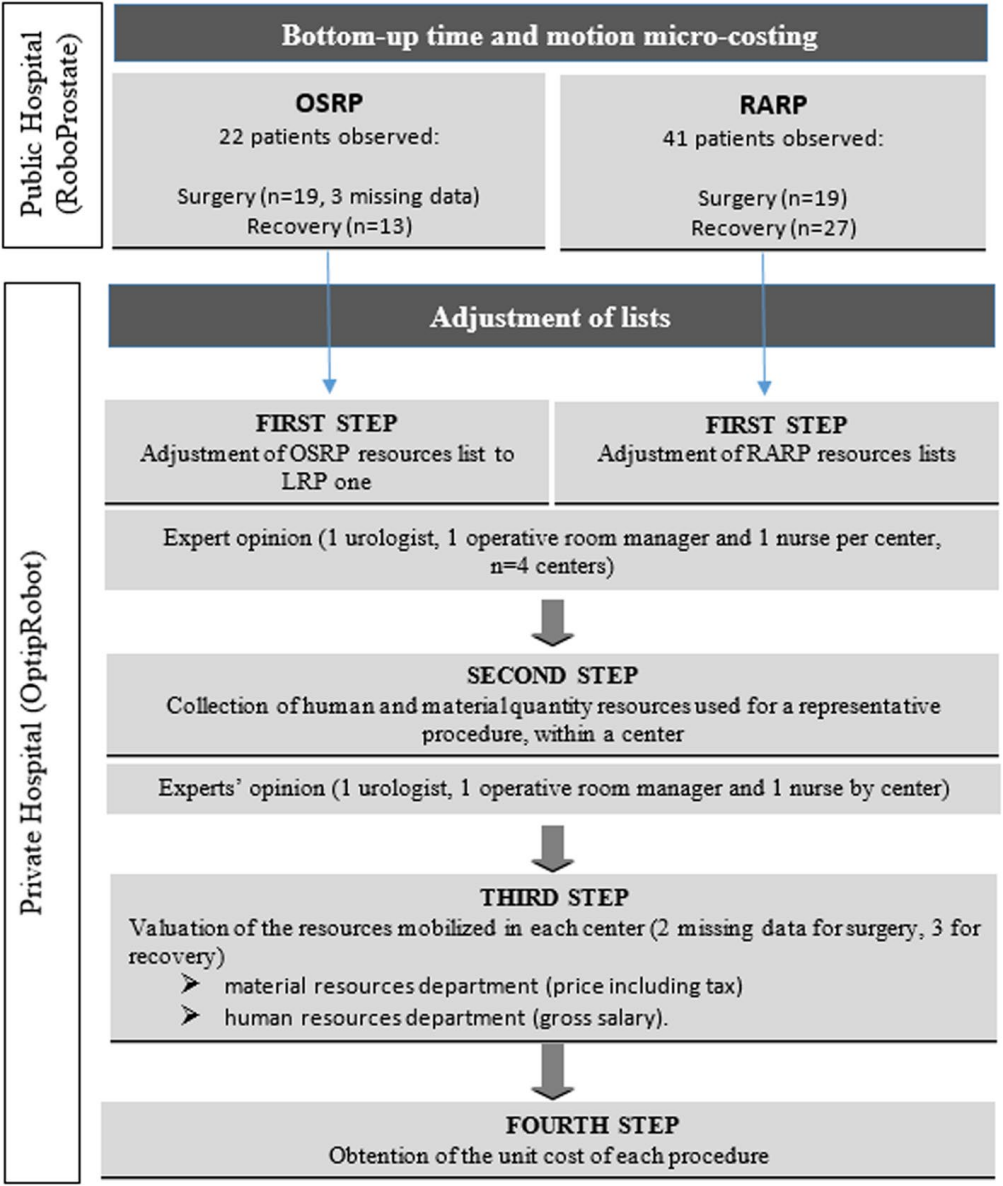
Data collection model

The study design is described in Fig. 1. This study is reported in accordance with the updated CHEERS guidelines [22].

### Micro-costing data collection

Micro-costing, was performed on observed data using a bottom-up, time-and-motion analysis from the RoboProstate study to compare RARP versus OSRP average cost from the perspective of public hospital. This study was carried out in two public hospitals: one included patients operated by RARP and the other by OSRP. Patients were not randomised but matched on age and AMICO’s risk class between groups. Separate case report forms were pre-established for surgery and recovery.

The OptiPRobot project estimated RARP versus LPR costs from the private hospital perspective (four centres) by adapting the materials and personnel lists from the RoboProstate study (provided experts’ declarative data).

Surgeons included in both studies had to meet a minimum experience threshold—at least 20 conventional laparoscopic and 10 robot-assisted prostatectomies performed personally—to reduce learning curve effects; no surgeon operated in both public and private centers.

### Patients

The RoboProstate study included men 45-75 years old with localised prostate cancer considering RP. To evaluate costs, 20 RPs per type of procedure were considered sufficient to reflect real-life case variability and the associated variation in costs, for surgery and recovery [23].

The estimated cost of the RP procedure is the sum of the cost of the Operative Room (OR) (from room preparation until cleaning and storage) plus recovery in the Recovery Room (RR). These is overlap since patients are transferred to RR before OR cleaning. Patients could be observed in one or both phases, with observations made depending on research staff availability. Thus, 19 surgeries and 13 recoveries were observed in OSRP from 22 patients; and 19 surgeries and 27 recoveries observed in RARP from 41 patients (Fig. 1).

### Valuation of resources and costs

Costs (personnel, materials) were valued in 2018 in euros from the perspective of the health establishment (cf. supplementary file1 for more details).

### Human resources

Personnel cost was based on the average gross annual salary in 2018 for each centre in each professional category, and on the number of hours worked per year, based on the collective agreements, accounting for on duty, on-call, or Sunday pay. Monthly salaries were transformed into average cost per minute, without distinction according to seniority or grade. For RoboProstate study, gross salary was obtained only for the coordinator centre.

#### Material resources

The unit costs were obtained from each centre’s purchasing software, including taxes. For single-use materials, the unit acquisition price including all taxes was used. For reusable materials, an amortized cost was estimated by considering (i) the acquisition price including taxes, (ii) the number of years of depreciation according to centre cost accounting rules and (iii) the number of surgeries performed per year.

The formula used was:

$$Amortized unit cost=\frac{Cost of aquisition including tax}{Number of years of amortisment*Number of surgeries per room per year}$$

The cost of acquisition of the Da Vinci robot was majored by the annual maintenance cost during the relevant amortization period. For reusable but limited material, the acquisition price was divided by its number of lives.

#### Total costs

The cost of surgery was calculated as the sum of the cost of medical equipment, excluding the robot, and medical staff time for the four periods (defined as (1) OR Preparation before patient's arrival, (2) Patient preparation for surgery, (3) Surgical time: from incision to closure and (4) After closure: Patient preparation for transfer + OR disassembly, storage and cleaning), majored by a 10.84% overhead representing the costs of structural, logistics and general management and sterilisation from the breakdown of costs for French DRG 12C111 (Major pelvic surgery in men for malignant tumours, level 1 in the National Cost Scale (NCS)). The robot amortized cost was added without overheads as maintenance costs were already accounted for.

Surgery cost was estimated by:

$$\text{SC }= [\text{Csmwr }+\sum_{T=1}^{4}Csp(t)]+ [0.1084*(\text{Csmwr }+\sum_{T=1}^{4}Csp(t))]+ Cr$$

Where SC = Surgery Cost, Csmwr = Cost of surgical material without robot, Csp(t) = Cost of surgical personal par period of surgery, T = period of surgery, and Cr = Amortized cost robot.

The cost for recovery corresponded to the sum of the cost of material and personnel, with a 1.99% overhead deduced by the same methodology but considering only structural costs. The recovery cost was estimated by:

$$\text{RC }= [\text{Crm }+\text{Crp}]+[0.0199 \left(\text{Crm }+\text{Crp}\right)]$$

Where RC = Recovery cost, Crm = Cost of recovery material, and Crp = Cost of recovery personal.

### Time horizon

The study considered only the costs associated with the RP procedure, divided into'surgery'(preparation of the OR until its cleaning) and'recovery'(patient arrival into RR until his discharge). Personnel costs was classified into four steps: 1. preparation of theatre material before patient arrival; 2. patient preparation; 3. surgical time: incision to closure; 4. after closure: preparing patient for transfer and disassembly, storage and cleaning of theatre.

### Analytical methods

For RoboProstate study, general characteristics, disease stage and health status were compared between patients operated by RARP versus OSRP and between patients observed or non-observed for the surgery and recovery estimates.

Descriptive statistics are reported as counts and percentages for categorical variables and means and standard deviations for continuous variables with normal distribution and median and quartiles for others. Comparisons were performed with Wilcoxon-Mann–Whitney, χ2, Student, or Fisher exact test as appropriate.

For both studies, average cost of each type of surgery and the difference is presented with 95% confidence intervals. The relative cost difference was estimated in percentage and compared by Student test.

To take into account the disequilibrium between the groups in the RoboProstate study, a mixed regression model with a random effect on the patient stratum (D'Amico classes and age classes) was performed.

In addition, the costs by expenditure item and the cost per minute was estimated.

All tests were two-tailed and conducted at alpha = 0.05 significance level. Statistical analysis was performed using SAS institute software, Cary, NC, USA version 9.3.

### Sensitivity analysis

A deterministic sensitivity analysis was conducted on the main factors: amortized cost of the robot and cost of the personnel. Low and high limit of the amortized cost of the robot was defined according to variations in activity and the purchase cost. Personnel cost was varied depending on the salary per minute, using gross annual salary ranges to deduce minimum and maximum cost per minute.

Supplementary Table 1 identifies the hypotheses and the corresponding variation values.

## Results

### Patient’s characteristics

Thirty-eight surgeries were observed in the RoboProstate study, 19 in each procedure. Patient clinical characteristics were compared between RARP versus OSRP and between the 38 observed patients versus the 52 non-observed. Patients in the RARP group were older, with a worse Gleason score at 1 month, and the d'Amico risk class differed between groups. The patients observed for surgery had a slightly smaller prostate volume than the non-observed patients, without statistical difference between the techniques. The other criteria were comparable (Table 1). Similar findings were seen for the patients observed for recovery (Table 2). Thus, an adjustment on the stratum (Amico risk class – age class) of the patient accounted for these potential imbalances.

**Table 1:** Characteristics of micro-costing surgery patients

	*RARP N=19*	*OSRP N=19*	*Observed surgery sample N=38*	*Non-observed surgery sample N = 52*
*General characteristics (no missing value)*				
Age, in years				
Mean ± StdDev	64.84 ± 4.97	61.42 ± 5.25	63.13 ± 5.33	64.9 ± 5.29
*p-value*	p=0.0465*		*p=0.1211*^*a*^	
BMI, in kg.cm-^2^				
Mean ± StdDev	26.05 ± 2.49	24.89 ± 3.1	25.47 ± 2.83	26.19 ± 3.55
*p-value*	*p=0.2124*^*a*^		*p=0.3042*^*a*^	
*Disease stage*				
Prostate volume, in cm3				
Median [IQR q1;q3]	42.5 [40; 53]	40 [30; 65]	42 [38; 55]	67 [39; 86]
Missing	7	8	15	19
*p-value*	*0.4113*^*b*^		*0.0228*^*b*^	
ISUP score at 1 month, %				
ISUP 1	3 (15.8%)	11 (57.9%)	14 (36.8%)	11 (22.4%)
ISUP 2	9 (47.4%)	8 (42.1%)	17 (44.7%)	17 (34.7%)
ISUP 3	5 (26.3%)	0	5 (13.2%)	13 (26.5%)
ISUP 4	2 (10.5%)	0	2 (5.3%)	6 (12.2%)
ISUP 5	0	0	0	2 (4.1%)
Missing	0	0	0	3
*p-value*	*0.0046*^c^		*0.166*^c^	
D'Amico risk class, %				
Low risk	5 (26.3%)	12 (63.2%)	17 (44.7%)	12 (23.1%)
Moderate risk	9 (47.4%)	5 (26.3%)	14 (36.8%)	24 (46.2%)
High risk	5 (26.3%)	2 (10.5%)	7 (18.4%)	16 (30.8%)
*p-value*	*0.0734*^c^		*0.088*^c^	
Total PSA at inclusion, in ng/ml				
Median [IQR q1;q3]	7.49 [5.39; 12.03]	6.78 [4.95; 9.7]	7.14 [5.36; 10.46]	7.8 [5.81; 10.72]
Missing	0	0	0	0
*p-value*	*0.3637*^*b*^		*0.4874*^*b*^	
T pathological stage, %				
1	0	2 (10.5%)	2 (5.3%)	0
2	14 (73.7%)	13 (68.4%)	27 (71.1%)	36 (72%)
3	5 (26.3%)	3 (15.8%)	8 (21.1%)	14 (28%)
X	0	1 (5.3%)	1 (2.6%)	0
Missing	0	0	0	2
p-value	0.4555^c^		0.2180^c^	
*Health Status*				
WHO Score, %				
Autonomy	17 (89.5%)	19 (100%)	36 (94.7%)	52 (100%)
Autonomy compatible with the activities	2 (10.5%)	0	2 (5.3%)	0
*p-value*	*0.4865*^c^		*0.1755*^c^	
ASA Score, %				
I	7 (41.2%)	5 (35.7%)	12 (38.7%)	24 (51.1%)
II	10 (58.8%)	7 (50%)	17 (54.8%)	19 (40.4%)
III	0	1 (7.1%)	1 (3.2%)	3 (6.4%)
IV	0	1 (7.1%)	1 (3.2%)	1 (2.1%)
Missing	2	5	7	5
*p-value*	*0.5620*^c^		*0.6034*^c^	
Adapted Charlson Score (Quan, 2010), %				
Score = 0	13 (68.4%)	11 (68.8%)	24 (68.6%)	41 (78.8%)
Score ≥1	6 (31.6%)	5 (31.3%)	11 (31.4%)	11 (21.2%)
Missing	0	3	3	0
*p-value*	*1*^c^		*0.3205*^c^	

^a^ T-test || ^b^ Wilcoxon-Mann-Whitney Test || ^c^Fisher Test

ASA: American Society of Anesthesiologists; PSA: Prostate Specific Antigen; ISUP: International Society of Urological Pathology.

**Table 2:** Characteristics of micro-costing recovery patients

	*RARP N=27*	*OSRP N=13*	*Micro-costing recovery sample N=40*	*No micro-costing recovery sample N = 50*
*General characteristics (no missing value)*				
Age, in years				
Mean ± StdDev	63.59 ± 4.96	59.38 ± 5.69	62.23 ± 5.51	65.7 ± 4.72
*p-value*	0.0217*		0.0018*	
BMI, in kg.cm-^2^				
Mean ± StdDev	26.79 ± 3.85	25.22 ± 2.12	26.28 ± 3.44	25.58 ± 3.12
*p-value*	0.1053*		0.3108*	
*Stage of disease*				
Prostate volume, in cm3				
Median [IQR q1;q3]	41 [30; 68.5]	40 [30; 70]	40 [30; 70]	60 [43; 75]
Missing	15	4	19	15
*p-value*	*0.8319*^*b*^		*0.0697*^*b*^	
Gleason score at 1 month, %				
ISUP 1	7 (26.9%)	8 (66.7%)	15 (39.5%)	10 (20.4%)
ISUP 2	7 (26.9%)	4 (33.3%)	11 (28.9%)	23 (46.9%)
ISUP 3	7 (26.9%)	0	7 (18.4%)	11 (22.4%)
ISUP 4	4 (15.4%)	0	4 (10.5%)	4 (8.2%)
ISUP 5	1 (3.8%)	0	1 (2.6%)	1 (2%)
Missing	1	1	2	1
p-value	0.0534^*c*^		0.2582^*c*^	
AMICO risk class, %				
Low class risk	7 (25.9%)	8 (61.5%)	15 (37.5%)	14 (28%)
Moderate class risk	11 (40.7%)	3 (23.1%)	14 (35%)	24 (48%)
High class risk	9 (33.3%)	2 (15.4%)	11 (27.5%)	12 (24%)
*p-value*	*0.1193*^*c*^		*0.4873*^*c*^	
Total PSA at inclusion, in ng/ml				
Median [IQR q1;q3]	7.8 [5.36; 10.74]	6.78 [5.16; 11]	7.65 [5.25; 10.87]	7.6 [5.85; 10]
*p-value*	*0.5477*^*b*^		*0.8107*^*b*^	
T pathological stage, %				
1	0	1 (7.7%)	1 (2.6%)	1 (2%)
2	20 (76.9%)	8 (61.5%)	28 (71.8%)	35 (71.4%)
3	6 (23.1%)	3 (23.1%)	9 (23.1%)	13 (26.5%)
x	0	1 (7.7%)	1 (2.6%)	0
Missing	1	0	1	1
p-value	0.2244^*c*^		0.8065^*c*^	
*Health Status*				
OMS Score, %				
Autonomie	26 (96.3%)	13 (100%)	39 (97.5%)	49 (98%)
Autonomie compatible avec les activités	1 (3.7%)	0	1 (2.5%)	1 (2%)
*p-value*	*1*^*C*^		*1*^*c*^	
ASA Score, %				
I	12 (50%)	2 (22.2%)	14 (42.4%)	22 (48.9%)
II	11 (45.8%)	6 (66.7%)	17 (51.5%)	19 (42.2%)
III	0	0	0	4 (8.9%)
IV	1 (4.2%)	1 (11.1%)	2 (6.1%)	0
Missing	3	4	7	5
*p-value*	*0.2881*^*c*^		*0.1015*^*c*^	
Adapted Charlson Score (Quan, 2010), %				
Score = 0	18 (66.7%)	9 (81.8%)	27 (71.1%)	38 (77.6%)
Score >= 1	9 (33.3%)	2 (18.2%)	11 (28.9%)	11 (22.4%)
Missing	0	2	2	1
*p-value*	*0.4520*^*c*^		*0.6199*^*c*^	

^a^ T-test || ^b^Wilcoxon-Mann-Whitney Test || ^c^Fisher Test

ASA: American Society of Anesthesiologists; PSA: Prostate Specific Antigen; ISUP: International Society of Urological Pathology.

### Incremental costs and outcomes

All costs estimations are reported in table 3.

**Table 3:** OSRP, RARP and LRP Costs estimations

	**Public hospital**		**Private Hospital**	
Expenditure	OSRP *n*=16 surgery and 13 recovery	RARP *n*=19 surgery and 27 recovery	RARP Center A and B *n* = 2 centers	LRP Center A and B *n* = 2 centers
Mean ± StdDev				
% of RP total cost				
RP total cost	1273 [1141.6; 1404.4]	5018.8 [4842.3; 5195.3]	6668.25€ (1 MD)	1984.9 (1 MD)
	100%	100%	100%	100%
Surgery total cost	1226.92 [1096.3; 1357.5]	4970.96 [4794.7; 5147.2]	6555.35 [4776.6; 8334.1]	1872 [1738.7; 2005.3]
	96.4%	99%	98.3%	94.3%
Recovery total cost	46.1 [31.8; 60.4]	47.8 [39.1; 56.5]	112.9 (1 MD)	112.9 (1 MD)
	3.4%	1%	1.7%	5.7%
**Time per procedure** mean [95 CI%]				
Total time of OR use: from preparation to storage/cleaning, in minutes	230.8 [205.9; 255.8] 1 MD	329.1 [302.5; 355.7] 1 MD	X	X
Surgery time (incision/closure), in minutes	162.6 [140.7; 184.5]	212.6 [186.2; 239]	228 [222.2; 233.8]	198 [114.7; 281.3]
**Surgery costs per procedure** mean [95% CI] % of surgery total cost				
Material surgery	241.33 [223.6; 259]	2270.4 [2221.3; 2319.5]	2349.35 [2062,9; 2635,8]	582.15 [546,8; 617,5]
	19.67%	45.7%	35.8%	31.1%
Staff costs, step 1 and 2	138.25 [117.8; 158.7] 11.27%	267.69 [235.8; 299.6] 5.4%	step 1 = 41.3 [8.6; 74] 0.6% step 2 = 194.6 [−16.8; 405.9] 3%	step 1 = 38.8 [1.1; 76.5] 2.1% step 2 = 130.53 [21.2; 239.9] 7%
Staff cost,step 3	661.88 [566.2; 757.5]	783.13 [646.7; 919.5]	1050.59 [894.4; 1206.8]	881.73 [596.6; 1166.8]
	53.95%	15.8%	16%	47.1
Staff cost, step 4	65.47 [47.5; 83.5]	68.35 [59.2; 77.5]	67.5 [20.7; 114.3]	55.73 [2.5; 108.9]
	5.34%	1.4%	1%	3.0
Amortized cost of robot and maintenance	0	1214	2450.63 [1484.9; 3416.3]	0
		24.4%	37.4%	0,00%
Overheads cost	119.99 [107.2; 132.8]	367.4 [350.2;384.6]	401.44 [321.9; 481]	183.08 [170.1; 196.1]
	9.78%	7.4%	6.1%	9.8
**Recovery costs** mean [95% CI] % of recovery total cost				
Staff cost	29.06 [19.2; 39]	26.35 [20.5; 32.2]	69.1	69.1
	63%	55.1%	61.2%	61.2%
Material cost	16.14 [8.9; 23.4]	20.49 [16.2; 24.8]	41.6	41.6
	35%	42.9%	36.8%	36.8%
Overheads cost	0.90 [0.62; 1.18]	0.93 [0.76; 1.1]	2.2	2.2
	2%	2%	2%	2%
**Costs per minute** mean [95% CI]				
RP total cost > per minute of OR use > per minute incision/closure	5.72 [−0.27; 11.7] 8.2 [7.5; 8.9]	15.42 [14.6; 16.2] 24.84 [22.6; 27.1]	X 29.2 *(1 MD)*	X 8.55 *(1MD)*
Surgery cost > per minute of OR use > per minute incision/closure	5.52 [5,2; 5,9] 7.9 [7,2; 8,6]	15.27 [14.5; 16] 24.62 [22.3; 26.9]	28.8 [20,2; 37,4]	9.85 [6,4; 13,3]
Recovery cost > per minute of OR use > per minute incision/closure	0.2 [0,14; 0,26] 0.28 [0,19; 0,37]	0.15 [0,12; 0,18] 0.22 [0,18; 0,26]	0.49 *(1 MD)*	0.47

#### RARP cost estimations

The average surgery cost was 4970.96€ [95% CI=4794.7; 5147.2] for RoboProstate study (public hospital) and 6555.35€ [95% CI=4776.6; 8334.1] for OptiPRobot study (private hospital:). For recovery, the average cost was 47.8€ [95% CI=39.1; 56.5] and 112.9€ respectively. The multivariate analysis made for public hospital did not change the results, with a cost of 4968.48€ [95% CI=4777.58; 5159.37] for surgery and 47.7€ [95% CI=33.7; 61.8] for recovery. The total cost was 5018.8€ [95% CI= 4842.3; 5195.3] for public hospitals and 6668.25€ for private. For both, the surgery cost represented more than 98% of the overall cost. Surgery times were similar: 212.6min [95% CI=186.2; 239] in public hospitals and 228 min [95% CI=222.2; 233.8] in private. Thus, estimated RP total cost per minute of surgery (incision/closure) was 24.84€ [95% CI=22.6; 27.1] for public hospital versus 29.2€ for private one.

The percentage distribution by expenditure item of surgery cost was similar in public and private hospitals, with a higher amortization cost of the robot in private clinics. This generated a difference in absolute surgery cost, estimated at +1585€ [95% CI=−203; +3372], representing an additional cost of 32% for the private hospital (Table 4). This difference is mainly due to the amortized cost of robot and maintenance: +1236.6 [95% CI=270.9; 2202.3], representing 78% of the surgery cost difference.

**Table 4:** OSRP, RARP and LRP Costs Differences

	**Cost difference**			
Expenditure item	RARP public versus RARP private Mean [95% CI] Variation relative, %	Public hospital: RARP - OSRP Mean [95% CI]		Private hospital: RARP - LRP Mean [95% CI] Variation relative, %
Mean [95% CI]				
% of RP total cost		*p*-value (Student)	Variation relative, %	
RP total cost	1650	3745.8 [3525.8; 3965.8]		4683.36 (1 MD)
	+33%	< 0.0001	+294%	+236%
Surgery total cost	1584.4 [−203; +3372]	3744.04 [3524.6; 3963.4]		4683.35 [2900; 6467.2]
	+32%	< 0.0001	+305%	+250%
Recovery total cost	65.1	1.7 [−15; 18.4]		0
	+136.2%	0.8387	+3.7%	0
Time per procedure mean +/- sd				
Total time of OR use: from preparation to storage/cleaning, in minutes	X	98.3 [63.3; 133.3] <0.0001 | +42.6%		X
Surgery time (incision/closure), in minutes	15.4 [−11.6; 42.4] +7.2%	50 [16.9; 83.1] 0.0055 | +30.8%		30 [−53.5; 113.5] +15.2%
Surgery costs per procedure mean [95% CI] % of surgery total cost				
Material surgery. mean ± sd	79 [−211.6; +369.6]	2029.1 [1976.9; 2081.3]		1767.2 [1478.6; 2055.8]
	5%	< 0.0001	+841%	+304%
Staff costs: step 1* and 2*	31.8 [−184.4; 248.1]	129.5 [91.5; 167.3]		Phase 1: 2.5 [−47.4; 52.4] +6.4%
	2%	< 0.0001	+93.6%	Phase 2: 64.03 [−174; 302] +49.1%
Staff cost: step 3*	267.5 [60.1; 474.8]	121.3 [−45.3; 287.8]		168.9 [−156.2; 493.9]
	16.9%	0.1783	+18.3%	+19.2%
Staff cost: step 4*	−0.85 [−48.6; 46.9]	2.88 [−17.3; 23.1]		11.77 [−59.1; 82.7]
	<0.1%	0.7821	+4.4%	+21.1%
Amortized cost of robot and maintenance	1236.6 [270.9; 2202.3]	1214		2450.6 [1484.9; 3416.3]
	78%	< 0.0001	x	x
Overheads cost	34 [−47.4; +115.4]	247.4 [225.9; 268.9]		218.4
	2%	x	x	+119.3%
**Recovery costs** mean [95% CI] % of recovery total cost				
Staff cost	42.75	−2.71 [−14.2; 8.8]		0
	+162%	0.6253	−9.3%	
Material cost	21.1	4.35 [−4.1; 12.8]		0
	+103%	0.2938	+27%	
Overheads cost	1.3	0.03 [−0.3; 0.36]		0
	+137%	x	x	
**Costs per minute** mean [95% CI]				
RP total cost > per minute of OR use > per minute incision/closure	x+0.06 (+0.2%)	+9.7 [8.8; 10.6] | x | +170%+16.7 [14.3; 19] | x | +203%		x16.35 (+191%)
Surgery cost > per minute of OR use > per minute incision/closure	x4.2 [−4.7; 13.1] (+17%)	+ 9.75 [8.9; 10.6]| < 0.0001 | +177%+ 16.72 [14.3; 19.1] | < 0.0001 | +212%		x18.95 [9.7; 28.2] (+192%)
Recovery cost > per minute of OR use > per minute incision/closure	x0.27 (+123%)	−0.05 [−0.12; 0.02] | 0.1315 | −25%−0.06 [−0.16; 0.04] | 0.1814 | −21.4%		x0.02 (+4.3%)

^*^ Step 1. preparation of theatre material before patient arrival; Step 2. patient preparation; Step 3. surgical time: incision to closure; Step 4. after closure: preparing patient for transfer and disassembly, storage and cleaning of theatre

#### RARP versus OSRP

RARP and OSRP costs were compared from the public hospital perspective. Multivariate analyses did not reveal any effect of patient stratum for the regression model of the cost of surgery (p=0.3883; ICC=2.9%) or recovery (p=0.2109; ICC=12.2%). Thus, results are presented as univariate.

The cost of OSRP was 1273€ [95% CI=1141.6; 1404.4] versus 5018.8€ [95% CI=4842.3; 5195.3] in RARP, the surgery accounting for 96.4% of the total cost for OSRP versus 99% for RARP (Table 3). RARP was longer than OSRP, with +98.3 [95% CI=63.3; 133.3] minutes for room occupancy (p<0.0001, +42.6%) and +50 [95% CI=16.9; 83.1] minutes surgical time in RARP (p=0.0055, +30.8%). This corresponded to an average extra cost of +9.7€ [95% CI=8.8; 10.6] per minute of OR use (+170%) and +16.7€ [95% CI=14.3; 19] per surgical minute (+203%) in RARP (Table 4).

Most surgical costs were personnel costs during the incision/closure in OSRP (54%), whereas the costs of the surgical material (46%) and the robot and its maintenance (24%) were dominant in RARP. The cost of recovery had little impact on the type of surgical technique (Table 3).

The cost difference per patient was significant (p<0.0001), with a mean extra cost of +3745.8€ [95% CI=3525.8; 3965.8], (+294%) per RP surgery. This difference is largely due to the cost of the surgical equipment (54%) and the robot (32%). In absolute values, the robot induced an additional cost per RP surgery of +2029€ for the surgical material and +1214€ for the depreciation cost of the robot (Table 4).

#### RARP versus LRP

The total estimated average cost was 6668.25€ for RARP and 1984.9€ for LRP, consisting of respectively 98.3% and 94.3% of surgery cost (Table 3).

RARP required an additional +30 min [95% CI=−53.5; 113.5], (+15.2%) versus LRP, corresponding to an extra surgery cost of +18.95€ [95% CI=9.7; 28.2], (+192%) per minute.

Again, the distribution of costs between the two techniques differed due to the robot extra cost. Thus, the main cost for LRP was for personnel during incision/closure (47%), whereas it was the cost of the robot (37.4%) and the surgical material (35.8%) in RARP (Table 3).

In absolute values, the estimated cost of RARP was over twice that of LRP, corresponding to an extra cost of +4683.36€. The extra cost is linked to the purchase and maintenance of the robot (+2450.6€ [95% CI=1484.9; 3416.3]) and the surgery equipment (+1767.2€ [95% CI=1478.6; 2055.8])(Table 4).

The recovery cost was 112.9€, irrespective of the type of surgery.

### Sensitivity analysis

#### RARP versus OSRP

In bi-variate analysis, whatever the variations in the cost of robot and salaries parameters, RARP was always more expensive than OSRP. This extra cost varied from +3179.5€ [95% CI=3050; 3309] with minimum parameter values to +4591.4€ [95% CI=4271.8; 4911] at maximum. The variation in the robot cost parameter had the greatest impact on the estimates. For a variation in the cost of the robot from 707.14€ to 1981.38€ per patient, the average RARP cost varied from 4512.2€ [95% CI=4335.7; 4688.7] to 5785.1€ [95% CI=5608.6; 5961.6], while the cost varied from 3690€ [95% CI=3555.9; 3824.1] to 3824.7€ [95% CI=3482.2; 4167.2] for personnel parameter variations.

The difference in average cost between OSRP versus RARP from the public hospital's perspective varied from +3239.2€ [95% CI=3019.2; 3459.2] to +4512.1€ [95% CI=4292.2; 4732] (incremental cost per patient with the robot), i.e., a relative increase of +255% to +354% for RARP versus OSRP for robot cost parameter variations.

This corresponds to an average overhead of +8.1€ [95% CI=7.4; 8.9] to +12.1€ [95% CI=11.1; 13.1] per OR minute, a relative increase of +142% to +212% for RARP compared to OSRP. The variation of robot amortized cost induced a variation with the mean cost around ±15% to 20% (Table 5).

**Table 5:** Sensitivity analysis for OSRP vs RARP and LRP vs RARP. OR=operative room

**Study**	**Parameter**	**Estimates**	**OSRP**	**RARP**	**Differences** **Mean [95% CI]**	**Relative difference, %** **between OSRP and RARP**	**Relative difference with basis mean difference**
RoboProstate: OSRP vs RARP	Amortized cost of robot and maintenance	Min value, 707.14€ per patient					
		Act cost, in €	1273 [1141.6; 1404.4]	4512.2 [4335.7; 4688.7]	3239.2 [3019.2; 3459.2]	+255%	−13.5%
		Act cost per minute of OR use	5.7 [5.4; 6.1]	13.9 [13.2; 14.5]	8.1 [7.4; 8.9]	+142%	−16.5%
		Act cost per surgical minute	8.2 [7.5; 8.9]	22.3 [20.3; 24.3]	14.4 [12; 16.2]	+173%	−13.8%
		Max value, 1980.38€ per patient					
		Act cost, in €	1273 [1141.6; 1404.4]	5785.1 [5608.6; 5961.6]	4512.1 [4292.2; 4732]	+354%	+20.5%
		Act cost per minute of OR use	5.7 [5.4; 6.1]	17.85 [16.9; 18.8]	12.1 [11.1; 13.1]	+212%	+24.7%
		Act cost per surgical minute	8.2 [7.5; 8.9]	28.7 [26; 31.4]	20.5 [17.7; 23.3]	+251%	+22.8%
	Cost of personnel	Salary MIN					
		Act cost, in €	883.5 [803.04; 963.9]	4573.5 [4466.2; 4680.8]	3690 [3555.9; 3824.1]	+418%	−1.5%
		Act cost per minute of OR use	3.98 [3.71; 4.25]	14.13 [13.3; 15]	10.2 [9.3; 11]	+255%	+5.2%
		Act cost per surgical minute	5.7 [5.2; 6.2]	22.8 [20.5; 25.1]	17.1 [14.7; 19.5]	+300%	+2.4%
		Salary MAX					
		Act cost, in €	1684.2 [1488.42; 1879.97]	5508.9 [5243.9; 5773.9]	3824.7 [3482.2; 4167.2]	+227%	+2.1%
		Act cost per minute of OR use	7.55 [7.05; 8.05]	16.9 [16.1; 17.7]	9.3 [8.4; 10.3]	+123%	−4.1%
		Act cost per surgical minute	10.8 [9.8; 11.8]	27.2 [24.9; 29.5]	16.4 [13.9; 18.9]	+151%	−1.8%
**Study**	**Parameter**	**Estimates**	**LRP**	**RARP**	**Differences** **Mean [95% CI]**	**Relative difference, % between LRP and RARP**	**Relative difference with basis mean difference**
OptiPRobot: LRP vs RARP	Amortized cost of robot and maintenance	MIN value					
		Act cost, in €	1984.9	4830.3	2845.4	+143%	−39.2%
		Act cost per minute of OR use	X	X	x	x	X
		Act cost per surgical minute	10.02	21.19	11.16	+111%	−31.7%
		MAX value					
		Act cost, in €	1984.9	7401.8	5416.9	+273%	+15.7%
		Act cost per minute of OR use	x	x	x	x	X
		Act cost per surgical minute	10.02	32.46	22.44	+224%	+12.9%
	Cost of personnel	Salary MIN					
		Act cost, in €	1660.61	6275.5	4614.9	+278%	−1.5%
		Act cost per minute of OR use	x	x	x	x	X
		Act cost per surgical minute	8.65	27.57	18.92	+219%	+15.7%
		Salary MAX					
		Act cost, in €	2309.2	7061.03	4751.8	+206%	+1.5%
		Act cost per minute of OR use	X	X	x	x	X
		Act cost per surgical minute	12.02	31.02	19	+159%	+16.2%

#### RARP versus LRP

In bi-variate analysis, whatever the variations in the cost robot and salaries parameters, RARP was always more expensive than LRP. This extra cost varied from +2776.9€ [95% CI=1839.8; 3714] for minimum parameter values to +5485.4€ [95% CI=3556.6; 7414.2] at maximum.

The amortized cost of the robot and maintenance ranged from 735.8€ to 3687€ per patient in centre 1 and from 489.5€ to 2681.4€ in centre 2. This variation induced a minimum difference cost of +2845.4€ (+143%) to +5416.9€ (+273%), corresponding to an extra cost per surgical minute of 11.16€ (+111%) to 22.44€ (+224%) (Table 5).

A salary increase of 25% had negligible impact on the cost difference, only increasing the cost difference by about 1.5% compared to the current average cost difference estimated at €4683.36.

The most influential parameter on the cost difference was the amortized cost of the robot (Table 5). Thus, the relative differences estimated between the cost difference in the main analysis and those estimated in the sensitivity analysis were strongest for the parameter of the amortized cost of the robot.

## Discussion

Our analysis revealed an additional cost for RARP of 1650€ for the private hospital perspective (+33%) compared to the public hospital. This was mainly due to the purchase price of the amortized cost of the robot and maintenance (+1237€; 78%). Private hospitals had higher purchase prices, different maintenance contracts and lower activity.

In some private participating centers, surgeons reported that an additional fee was required for the use of the robot, which they had to personally cover. When such fees were variable or not transparently defined, they could influence the decision to perform robotic surgery. This highlights the importance of stable and predictable professional fee structures, particularly in systems where surgeon remuneration is decoupled from institutional pricing.

The estimated amortized robot price per act varies between studies from 3456€ in 2011 based on 5 years of amortization and only 70 acts/year [18], to 2422€ in 2016 [20], 2571€ in 2019 [17] and 1246€ in 2021 [16]. Our estimates of 1214€ for the public hospital and 2451€ for private reflect the potential changes in this cost item. Robot use should be optimised to reduce its amortized cost per procedure.

It is difficult to compare our estimated costs against other studies because expenditure items and estimation methods differ. Nevertheless, it is possible to compare the equipment expenditure, even though we accounted for all equipment, whereas other articles considered the surgical equipment alone. Thus, the estimated cost of the RARP material ranges from 1311€ to 3234€ [16–20], varying over time, between countries, and depending on the items considered. Since we considered all material in our estimate, the cost of the surgical and anaesthetic material thus amounts to 2270€ for the public hospital and 2350€ for private.

Bolenz et al., Linderberg et al. and Labban et al. estimated the cost of the LRP specific material at 483€, 2418€ and 1585€ respectively against 582€ in our study. For the OSRP technique, the estimates were respectively 123€, 91€ and 743€, against 241€ in our study. Finally, only Lindenberg et al. provided details of the personnel cost, with an estimate of 1036€ for the RARP technique, compared with 1107€ in our study, and 1225€ for the LRP technique, compared with 1119€ (public) and 1355€ (private) in our study [17]. These limited data suggest that personnel costs are more stable across studies.

A strength of the study is the quality of the observed data from a public hospital. Micro-costing is the gold-standard, allowing for maximum variability between patients and techniques [24]. Another was identifying expense items (four phases of surgery, surgical equipment, robot, recovery) to determine targets for price decreases. One limitation is the small number of centres providing complete data. Nevertheless, RP is highly standardised and the micro-costing data were consistent.

Our study confirms the economic levers linked to RARP procedures: the costs of acquiring the robot, the activity and the specific surgical material [11, 21]. The lower acquisition price of the robot and consumables once the current robot monopoly ends could significantly reduce these costs [25]. Additionally, the"Extended Use Program"increases the lifetime of several high-volume instruments from 10 to 12-18 uses [2], further lowering the cost.

Future French and European studies can use our data for longer term estimations and to highlight the economic and social impact of the Da Vinci robot. Such studies are needed to determine possible reimbursement of the additional costs.

## Conclusion

Robot-assisted surgery was implemented in France before evaluation. This study lays the groundwork for an economic evaluation of the Da Vinci robot in RP by assessing the incremental costs compared to OSRP or RARP and identifies mechanisms to reduce them. The emergence of new robotic systems (e.g., Hinotori, Hugo RAS, MicroPort) raises important future research perspectives, particularly in comparing their early cost profiles to existing data from established platforms like Da Vinci. We estimated the incremental cost of using the Da Vinci robot for prostatectomy to conduct further analysis and estimate potential offsetting economic effects from a medium- to long-term perspective. Longer term analysis using our cost data will provide better estimates of the residual incremental costs.

## Supplementary Information

Supplementary Material 1.

## Data Availability

The datasets analyzed during the current studies are available from the corresponding author on reasonable request.

## Abbreviations

CNIL: Commision Nationale de l’Informatique et des Libertés
DRG: Diagnosis Related Group
GDPR: General Data Protection Regulation
LRP: Laparoscopic Radical Prostatectomy
OR: Operative Room
OSRP: Open Surgical Radical Prostatectomy
RARP: Robotic assisted Radical Prostatectomy
RP: Radical Prostatectomy
RR: Recovery Room
