# Supplementary material for: Robot-assisted approach versus open surgery and conventional laparoscopy for radical prostatectomy for prostate cancer: a micro-costing study
Source: Health Econ Rev. 2025 Jul 5;15:55. doi: 10.1186/s13561-025-00652-5 (PMC12228392; doi:10.1186/s13561-025-00652-5)
Supplement: Supplementary file 1 — Supplementary Material 1. [file 13561_2025_652_MOESM1_ESM.docx]

|  | | RoboProstate Study – Public Hospital | | | OptiProbot study – Private Hospital | | |
| --- | --- | --- | --- | --- | --- | --- | --- |
| Parameter | | Ref value | Low value | High value | Ref value | Low value | High value |
| Amortized cost of robot and maintenance | Hypothesis | Real activity (n=233/yr) and cost acquisition | Maximum of robot activity (n=400/yr) | New robot model acquisition | Real activity and cost acquisition | Doubling of the activity + acquisition price divided by 2 | New robot model acquisition |
|  | Cost per patient | 1214€ | 707.14€ | 1980.38€ | C1 =2943.4€  C2 =1957.9€ | C1=735.8€  C2=489.5€ | C1=3687€  C2=2681.4€ |
| Cost of personnel | Hypothesis | Mean salary estimate | Minimum salary estimate | Maximum salary estimate | Mean salary estimate | Mean salary estimate  -25% | Mean salary estimate +25% |

Supplementary Table 1: Variation of parameters for sensitivity analysis
